# Healthcare-seeking behavior for children aged 0–59 months: Evidence from 2002–2017 Indonesia Demographic and Health Surveys

**DOI:** 10.1371/journal.pone.0281543

**Published:** 2023-02-09

**Authors:** Uswatun Khasanah, Ferry Efendi, Eka Mishbahatul M. Has, Qorinah Estiningtyas Sakilah Adnani, Kadar Ramadhan, Yessy Dessy Arna, Wedad M. Almutairi

**Affiliations:** 1 Faculty of Nursing, Universitas Airlangga, Surabaya, Indonesia; 2 Faculty of Medicine, Universitas Padjadjaran, Bandung, Indonesia; 3 Department of Midwifery, Poltekkes Kemenkes Palu, Palu, Indonesia; 4 Center for Stunting Studies, STBM and Disaster Health, Poltekkes Kemenkes Palu, Palu, Indonesia; 5 Politeknik Kesehatan Kementerian Kesehatan, Surabaya, Indonesia; 6 Faculty of Nursing, Maternity and Childhood Department, King Abdulaziz University, Jeddah, Saudi Arabia; Flinders University, AUSTRALIA

## Abstract

**Background and objective:**

Healthcare-seeking behavior for children is crucial for reducing disease severity. Such behavior can improve child health outcomes and prevent child morbidity and mortality. The present study sought to analyze the determinants of mothers’ engagement in healthcare-seeking behavior for children with common childhood diseases, focusing on mothers of children aged 0–59 months in Indonesia.

**Methods:**

This cross-sectional study comprised a secondary data analysis using the 2002–2017 Indonesia Demographic and Health Survey (IDHS) databases. We included all women surveyed aged 15–49 years old who had children under five years of age. We weighted the univariate, bivariate, and multivariate logistic regression analysis of healthcare-seeking behavior for children aged 0–59 months.

**Results:**

We analyzed data for 24,529 women whose children were under five years of age at the time of survey. Common diseases, such as diarrhea, fever, and acute respiratory infection (ARI) were the most frequently cited reasons for healthcare-seeking behavior. During 2002–2017, the proportion of mothers seeking healthcare for their children with diarrhea increased from 67.70% to 69.88%, that with fever increased from 61.48% to 71.64% and that ARI increased from 64.01% to 76.75%. Multivariate analysis revealed that child’s age, child’s birth order, mother’s education, ability to meet expenses, distance to nearest healthcare facility, wealth index, place of residence, and region of residence, were significantly associated with healthcare-seeking behavior.

**Conclusion:**

Various individual and environmental-level factors influence healthcare-seeking behavior for childhood diseases. Available, accessible, and affordable health service facilities are recommended to assist socio-economically and geographically disadvantaged families.

## Introduction

Over the last two decades, significant progress has been made in regard to reducing morbidity and mortality worldwide [1]. Globally, mortality rates for children under five years of age have decreased, falling from 76 per 1,000 live births in 2000 to 39 per 1,000 live births in 2018 [2]. However, most deaths among children under five occur in developing countries, such as those in Sub-Saharan Africa and Central and Southern Asia. In 2019, countries from these regions accounted for approximately 80% of the 5 million deaths of children aged under five occurring globally [3]. Therefore, to achieve the United Nations Sustainable Development Goal of reducing the child mortality rate to ≤25 deaths per 1,000 live births by 2030, developing countries should be specifically targeted [4]. Notably, in most cases of deaths among children under five years of age the causes are either treatable or preventable, particularly when diagnosis and/or treatment occurs early [1].

Indonesia has approximately 270 million people, including up to 22 million children aged under five years, and has up to four million childbirths per year [5]. Despite a reduction from 97 deaths per 1,000 live births in 1991 to 32 deaths per 1,000 live births in 2015, Indonesia’s mortality rate for children under five years remains higher than that of other Southeast Asian countries [6, 7]. Therefore, to prevent and reduce morbidity and mortality among this age group, it is essential that children receive appropriate and timely healthcare.

In Indonesia, acute respiratory infections (ARIs), diarrhea, and fever are recognized as the primary causes of morbidity and mortality among children under five years of age [8]. Indonesian national statistics have reported that approximately 4%, 14%, and 31% of children under five experience ARI symptoms, diarrhea, and fever, respectively [9, 10]. Notably, these percentages are similar to those recorded in countries deemed by the United States Agency for International Development to be priority areas: in countries in Western, Central, Eastern, and Southern Africa, 5.8%, 15.5%, and 23.4% of children under five years of age experience ARI symptoms, diarrhea, and fever, respectively [11]. Indonesian national data report that approximately 8% of children under five who experience ARI symptoms do not receive healthcare [9]. Studies have revealed that parents’ healthcare-seeking behaviors for their sick children has an important impact on treatment quality and the speed of the reduction of morbidity and mortality in children [11]. Furthermore, a systematic review showed that caregivers’ ability to recognize when healthcare is needed to treat common childhood illnesses is also critical for reducing child mortality in low- and middle-income countries [12]. Failure of caregivers and parents to seek appropriate care for sick children contributes to the high rates of under-five mortality in countries such as Ghana, Kenya, and Zambia [13].

Healthcare-seeking behavior is an effort to seek healthcare or medical treatment from healthcare providers in healthcare facilities [6]. Numerous complex factors influence parents’ decisions regarding whether to access healthcare for children under five years, including cultural beliefs and perceptions of disease, the perceived severity of the disease and the efficacy of treatment, area of residence (urban/rural), gender, household income/household wealth quintile, treatment costs, child’s age, household size, mother’s age, and mother’s education level [14, 15]. Among caregivers in Ghana, Kenya, and Zambia, education level, residence, and wealth index, as well as the distance to the nearest healthcare facility, have been shown to influence their decisions to seek treatment [13].

In the Indonesian setting, there are disparities between urban and rural areas regarding the quality and availability of healthcare facilities and services. Ensuring health-service equity within health systems and adequate utilization by parents and families remain the main challenges [16]. However, research on factors that influence healthcare-seeking behaviors among mothers in Indonesia, especially for children aged 0–59 months, remains scarce. The present study aimed to analyze the determinants of healthcare-seeking behaviors by mothers on behalf of their under-five children who have contracted common diseases. The current study, using nationally representative data on Indonesian households’ healthcare-seeking for the period of 2002–2017, may expand understanding of such determinants. This study provides vital insights into healthcare-seeking behavior, and key information that may guide policymakers in the area of maternal and child health.

## Materials and methods

### Design and data source

This present study comprised a secondary data analysis of nationally representative data from the Indonesian Demographic and Health Survey (IDHS) for the period of 2002–2017. The IDHS is a cross-sectional survey that is typically conducted every five years. The survey uses standardized international questionnaires and rigorous methods for data collection. Ethical approval for the IDHS was granted by the Inner-City Fund (ICF) OCR Macro (number 45 CFR 46) and the National Board Review of the Ministry of Health, Republic of Indonesia, and all participants provided informed consent prior to the study. Trained fieldworkers collected data through face-to-face interviews with women aged 15–49 years who had children aged 0–59 months at the time of the survey. A women’s questionnaire and a household questionnaire were used. The survey design employed a two-stage stratified cluster-sampling approach to ensure the representativeness of the data. The first stage of sampling was the selection of several census blocks using a probability proportional to size approach. In the second sampling stage, a random sample of ordinary households was selected from the list of census blocks [9]. The IDHS provides rich information and up-to-date estimates of fundamental demographic and health indicators, with a 98% response rate at the household level. Incomplete or unavailable data were excluded from the database. The study population was all women aged 15–49 years who had children under 5 years (0–59 months). All women aged 15–49 years were eligible for inclusion in the study. The final unit data comprised 24,529 mothers aged 15–49 who had children under five years (0–59 months). ICF International approved the use of the data for research purposes.

### Variables

#### Dependent variable

In this study, the outcome variable was mothers’ engagement in healthcare-seeking behavior for their children aged 0–59 months. Engagement in healthcare-seeking behavior was categorized as “no” or “yes.” For this analysis, “no (healthcare)” was defined as mothers seeking care from non-healthcare professionals or untrained health workers, or not seeking any healthcare, for their children under five. Meanwhile, “yes (health care)” was defined as mothers seeking care from healthcare workers or qualified and professional healthcare providers, such as doctors, nurses, and midwives in any healthcare facility, including hospitals, clinics, healthcare centers, and private healthcare practices.

#### Independent variable

In this study, independent variables included child factors such as child’s age, child’s gender, presence of diarrhea, fever, and/or ARI symptoms; maternal and paternal factors such as birth order, mother’s and father’s ages, mother’s and father’s education level and occupations, and area of residence; and socioeconomic/household factors such as whether the family were able to meet expenses, distance to the nearest healthcare facility, whether the family was covered by health insurance (yes/no), and household wealth index. Child age was classified into three categories: “< 12 months,” “12–36 months,” and “> 36 months,” respectively. The sex of each child was categorized as “female” or “male.” “Yes” and “no” answers were used to determine whether the children had diarrhea, fever, and/or ARI symptoms. Birth order was classified into three categories: “1st,” “2^nd^–3^rd^,” “≥4th.” Parental education was classified into four categories: no education, primary education, secondary education, and higher education. Maternal age was categorized into three categories: “15–24,” “25–34,” and “35–49,” while paternal age was categorized into four categories: “15–24,” “25–34,” “35–49,” and “≥ 50”. Parental occupation was classified as “working” or “not working.” Several indicators were used to determine household factors. Having enough money to meet expenses and the distance to the nearest healthcare facility were set as either “a big problem” or “not a big problem.” Health insurance coverage was categorized into two categories: “no” and “yes.” Following the DHS guidelines [17], families’ socioeconomic status was calculated using the household wealth index, and categorized into five classifications: “poorest,” “poor,” “middle,” “richer,” and “richest.” Factors associated with area of residence included place and region of residence. Place of residence was classified as “urban” or “rural,” while region was classified as “Eastern,” “Central,” or “Western” Indonesia, which represent the three primary geographical regions in Indonesia. The western region comprises Aceh, Bengkulu, Jambi, Lampung, North Sumatra, Riau, South Sumatra, West Sumatra, Riau, Bangka Belitung Islands, Banten, Jakarta, West Java, Central Java, Special Region of Yogyakarta, East Java, West Kalimantan and Central Kalimantan; the central region comprises South Kalimantan, East Kalimantan, North Kalimantan, North Sulawesi, Gorontalo, Central Sulawesi, West Sulawesi, South Sulawesi, Southeast Sulawesi, Bali, West Nusa Tenggara, and East Nusa Tenggara; and the eastern region comprises Maluku, North Maluku, West Papua, and Papua [18].

### Statistical analysis

Data were analyzed using STATA version 16 (Stata Corporation, College Station, Texas, USA). We calculated frequencies, proportions, and odds ratios (ORs) with 95% confidence intervals (CIs). We weighted the univariate, bivariate, and multivariate logistic regression analyses of mothers’ healthcare-seeking behaviors for their children aged 0–59 months. Chi-square statistical test and logistic regression was employed to examine the determinants of mothers’ engagement in healthcare-seeking behavior for children with common childhood diseases. For data from the four survey rounds, each round was equally weighted. Weighting was performed to consider the complex nature of the IDHS data, while the “svyset or survey estimation” command was used to adjust for the survey sampling method [19, 20]. The primary sampling unit or cluster variable, the stratification variable, and the weight variable were the three essential information to apply the complex sample design [20]. A likelihood ratio test was used to determine whether a factor was included in the model. A p-value of < 0.05 was interpreted as indicating statistical significance.

## Results

### Respondent characteristics

A total of 24,529 mothers with children under five years of age were included in the study. Table 1 shows the respondents’ characteristics. Overall, 66.45% of the mothers had sought healthcare for their children. According to the results, in the two weeks preceding the survey 28.56% of the children had experienced diarrhea, 68.86% had experienced fever, and 18.1% had shown ARI symptoms. Of the total children, 58.70% were aged 12–36 months, 51.98% were male, and 51.24% were 2^nd^ or 3^rd^ children. Over half of the mothers and fathers had completed secondary education. Half of the mothers (49.68%) and almost all of the fathers (99.06%) were working. Furthermore, most respondents (79.80%) reported no great difficulties meeting expenses and no great difficulties regarding the distance to the nearest healthcare facility (86.98%). Over half of the respondents (53.46%) were not covered by health insurance. In terms of household wealth index, 21.96% of the respondents were classified as the poorest. In addition, over half of the respondents (53.70%) were from rural areas, and most respondents (79.61%) were from Western Indonesia.

**Table 1:** Characteristics of mothers aged 15–49 years in Indonesia (n = 24,529; prepared by the authors from analysis of the data).

Variables	Total		2002		2007		2012		2017	
	(n = 24,529)	%^a^	(n = 3,815)	%^a^	(n = 5,995)	%^a^	(n = 8094)	%^a^	(n = 6625)	%^a^
**Child’s age (months)**										
< 12	4738	19.32	703	18.72	1196	19.39	1621	20.58	1218	18.05
12–36	14495	58.70	2512	64.51	3754	62.80	5137	63.16	3092	46.47
> 36	5296	21.98	600	16.77	1045	17.81	1336	16.26	2315	35.47
**Child’s sex**										
Male	13033	51.98	2008	51.16	3156	50.73	4341	52.92	3528	52.37
Female	11496	48.02	1807	48.84	2839	49.27	3753	47.08	3097	47.63
**Birth order**										
1^st^	7184	32.56	1076	29.01	1647	31.37	2594	35.96	1867	31.39
2^nd^–3^rd^	12432	51.24	1859	50.69	2979	50.61	4028	49.15	3566	54.66
≥ 4^th^	4913	16.20	880	20.29	1369	18.02	1472	14.89	1192	13.95
**Mother’s age (years)**										
15–24	5732	24.04	1040	28.30	1491	25.83	1893	23.76	1308	20.48
25–34	13041	52.58	2036	62.34	3189	52.75	4355	53.28	3461	51.70
35–49	5756	23.38	739	19.37	1315	21.43	1846	22.96	1856	27.82
**Mother’s education level**										
No education	672	2.18	150	3.80	223	3.07	206	1.76	93	1.02
Primary	8422	35.17	1757	48.15	2430	41.20	2500	31.12	1735	27.73
Secondary	12721	52.35	1699	43.17	2934	47.86	4350	55.30	3738	57.70
Higher	2714	10.30	209	4.88	408	7.86	1038	11.82	1059	13.55
**Mother’s occupation**										
Not working	11935	50.32	2118	59.38	3005	51.41	3649	46.19	3163	49.41
Working	12594	49.68	1697	40.62	2990	48.59	4445	53.81	3462	50.59
**Husband’s age (years)**						l				
15–24	1812	6.77	298	6.99	457	7.07	607	6.67	450	6.39
25–34	11911	48.33	1981	52.00	2901	47.33	3997	49.42	3032	45.81
35–49	10064	41.89	1405	37.59	2419	41.96	3284	41.05	2956	45.27
≥ 50	742	3.01	131	3.42	218	3.64	206	2.76	187	2.54
**Husband’s education level**										
No education	481	1.63	88	1.93	168	2.81	143	1.20	82	0.98
Primary	8350	35.82	1614	46.81	2298	39.97	2587	33.01	1851	29.56
Secondary	13120	52.48	1849	44.83	3051	48.20	4422	54.62	3798	57.82
Higher	2578	10.07	264	6.43	478	9.02	942	11.17	894	11.64
**Husband’s occupation**										
Not working	258	0.94	90	2.54	0	0.00	115	1.11	53	0.66
Working	24271	99.06	3725	97.46	5995	100.00	7979	98.89	6572	99.34
**Diarrhea**										
No	17598	71.44	3010	75.30	4554	76.93	5695	70.99	4339	65.12
Yes	6931	28.56	805	24.70	1441	23.07	2399	29.01	2286	34.88
**Fever**										
No	7476	31.14	1068	27.44	2001	35.58	2946	37.20	1461	21.94
Yes	17053	68.86	2747	72.56	3994	64.42	5148	62.80	5164	78.06
**Acute respiratory infection**										
No	19518	81.90	2514	70.56	3950	69.05	7171	89.83	5883	89.54
Yes	5011	18.10	1301	29.44	2045	30.95	923	10.17	742	10.46
**Meeting expenses**										
Big problem	5451	20.20	1107	25.15	1960	28.52	1295	14.70	1089	17.03
Not a big problem	19078	79.80	2708	74.85	4035	71.48	6799	85.30	5536	82.97
**Distance to nearest healthcare facility**										
Big problem	3755	13.02	657	13.04	1259	17.48	1033	10.90	806	11.76
Not a big problem	20774	86.98	3158	86.96	4736	82.52	7061	89.10	5819	88.24
**Covered by health insurance**										
No	7340	53.46	N/A	N/A	N/A	N/A	4828	62.99	2512	41.78
Yes	7379	46.54	N/A	N/A	N/A	N/A	3266	37.01	4113	58.22
**Wealth index**										
Poorest	7410	21.96	1217	21.78	1916	23.53	2388	21.30	1889	21.51
Poorer	5130	20.56	788	21.01	1242	20.07	1717	19.86	1383	21.60
Middle	4460	20.61	632	20.49	1054	20.59	1486	20.23	1288	21.16
Richer	4142	20.08	630	20.74	966	18.29	1392	20.90	1154	20.23
Richest	2387	16.79	548	15.99	817	17.52	111	17.71	911	15.49
**Place of residence**										
Urban	10526	46.30	1600	48.30	2168	41.17	3610	48.61	3148	46.75
Rural	14003	53.70	2215	51.70	3827	58.83	4484	51.39	3477	53.25
**Region of residence**										
Eastern Indonesia	1847	2.07	N/A	N/A	547	2.28	699	2.23	601	2.86
Central Indonesia	8303	18.32	1365	17.58	2080	19.42	2667	18.24	2191	17.87
Western Indonesia	14379	79.61	2450	82.42	3368	78.29	4728	79.53	3833	79.27
**Engagement in healthcare-seeking behaviors**										
No	8507	33.55	1639	42.54	2032	33.63	2640	31.09	2196	31.49
Yes	16022	66.45	2176	57.46	3963	66.37	5454	68.91	4429	68.51

^a^ Weighted proportion

**Fig 1:**
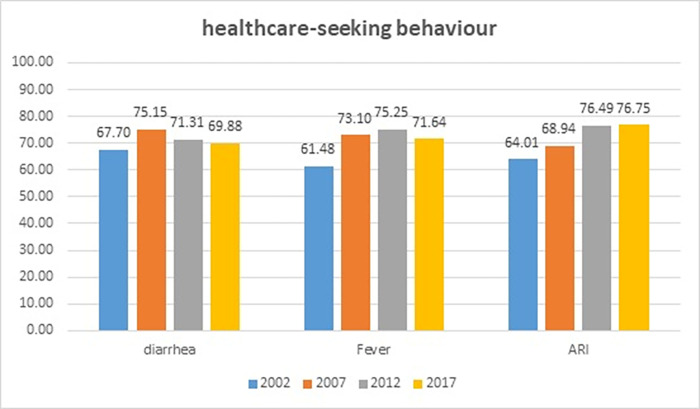
Mothers’ healthcare-seeking behavior for their children 0–59 months in Indonesia (prepared by the authors from the analysis of data).

The healthcare-seeking behaviors of the mothers are shown in Fig 1. The proportion of mothers who sought healthcare when their children had diarrhea increased from 67.70% in 2002 to 69.88% in 2017. Similarly, the proportion of mothers who sought healthcare when their children had a fever increased from 61.48% in 2002 to 71.64% in 2017. An increase was also observed among mothers who sought healthcare when their children had ARI symptoms, rising from 64.01% in 2002 to 76.75% in 2017.

### Bivariate analysis

The results of bivariate analysis are shown in Table 2. This analysis revealed that child’s age, birth order, mother’s education level and occupation, husband’s education level, ability to meet expenses, distance to the nearest healthcare facility, household wealth index, and place and region of residence were significantly associated with mothers’ engagement in healthcare-seeking behavior. Furthermore, significant associations with mothers’ engagement in healthcare-seeking behavior were also observed for children who experienced fever, diarrhea, and ARI symptoms. However, child’s sex, mother’s age, husband’s age and occupation, and health-insurance coverage were not significantly associated with mother’s engagement in healthcare-seeking behavior.

**Table 2:** Bivariate analysis of mothers’ engagement in healthcare-seeking behaviors for their children aged 0–59 months in Indonesia (prepared by the authors from the analysis of the data).

Variables	TOTAL						2002						2007						2012						2017					
	No		Yes		χ^2^	p-value	No		Yes		χ^2^	p-value	No		Yes		χ^2^	p-value	No		Yes		χ^2^	p-value	No		Yes		χ^2^	p-value
	n	%	n	%			n = 1639	%	n = 2176	%			n = 2032	%	n = 3963	%			n = 2640	%	n = 5454	%			n = 2196	%	n = 4429	%		
**Child’s age (months)**																														
< 12	1603	30.62	3135	69.38	56.67	<0.001***	296	39.30	407	60.70	4.84	0.514	377	28.53	819	71.47	33.57	0.001***	520	28.70	1101	71.30	18.23	0.009**	410	30.22	808	69.78	24.46	<0.001***
12–36	4844	33.06	9651	66.94			1063	42.82	1449	57.18			1237	33.37	2517	66.63			1617	30.67	3520	69.33			927	29.11	2165	70.89		
> 36	2060	37.45	3236	62.55			280	45.08	320	54.92			418	40.08	627	59.92			503	35.76	833	64.24			859	35.26	1456	64.74		
**Child’s sex**																														
Male	4460	33.25	8573	66.75	1.13	0.459	854	42.70	1154	57.30	0.04	0.910	1069	34.12	2087	65.88	0.66	0.587	1370	29.88	2971	70.12	6.23	0.061	1167	31.54	2361	68.46	0.01	0.947
Female	4047	33.89	7449	66.11			785	42.38	1022	57.62			963	33.12	1876	66.88			1270	32.46	2483	67.54			1029	31.44	2068	68.56		
**Birth order**																														
1^st^	2301	31.74	4883	68.26	58.83	<0.001***	430	38.89	646	61.11	25.72	0.041*	492	32.03	1155	67.97	19.06	0.029*	776	29.12	1818	70.88	17.44	0.012**	603	31.51	1264	68.49	0.32	0.902
2^nd^–3^rd^	4215	33.10	8217	66.90			755	41.54	1104	58.46			989	32.60	1990	67.40			1287	31.13	2741	68.87			1184	31.29	2382	68.71		
≥ 4^th^	1991	38.63	2922	61.37			454	50.27	426	49.73			551	39.28	818	60.72			577	35.74	895	64.26			409	32.26	783	67.74		
**Mother’s age (years)**																														
15–24	2003	33.66	3729	66.34	3.66	0.467	458	43.43	582	56.57	4.15	0.522	492	33.40	999	66.60	1.85	0.731	622	30.64	1271	69.36	1.29	0.733	431	30.72	877	69.28	0.94	0.743
25–34	4406	33.08	8635	66.92			847	41.09	1189	58.91			1040	33.10	2149	66.90			1378	30.84	2977	69.16			1141	31.37	2320	68.63		
35–49	2098	34.51	3658	65.49			334	45.17	405	54.83			500	35.20	815	64.80			640	32.15	1206	67.85			624	32.28	1232	67.72		
**Mother’s education level**																														
No education	350	48.05	322	51.95	129.63	<0.001***	92	52.78	58	47.22	73.83	<0.001***	112	49.58	111	50.42	45.22	0.001***	97	39.43	109	60.57	21.15	0.038*	49	52.53	44	47.47	30.76	<0.001***
Primary	3325	36.67	5097	63.33			891	48.35	866	51.65			950	36.50	1480	63.50			893	33.02	1607	66.98			591	30.61	1144	69.39		
Secondary	3972	30.87	8749	69.13			604	37.10	1095	62.90			860	31.03	2074	68.97			1324	29.11	3026	70.89			1184	30.22	2554	69.78		
Higher	860	33.48	1854	66.52			52	25.44	157	74.56			110	28.11	298	71.89			326	34.05	712	65.95			372	37.16	687	62.84		
**Mother’s occupation**																														
Not working	4014	32.30	7921	67.70	17.62	0.008***	828	39.53	1290	60.47	20.66	0.017**	970	31.92	2035	68.08	8.30	0.091	1162	30.33	2487	69.67	1.91	0.336	1054	30.04	2109	69.96	6.32	0.067
Working	4493	34.83	8101	65.17			811	46.94	886	53.06			1062	35.44	1928	64.56			1478	31.75	2967	68.25			1142	32.91	2320	67.09		
**Husband’s age (years)**																														
15–24	643	34.95	1169	65.05	5.95	0.482	124	39.11	174	60.89	1.48	0.935	153	32.76	304	67.24	4.18	0.682	208	36.48	399	63.52	11.07	0.162	158	32.53	292	67.47	5.56	0.307
25–34	4034	33.24	7877	66.76			839	42.59	1142	57.41			940	34.23	1961	65.77			1257	29.85	2740	70.15			998	30.95	2034	69.05		
35–49	3525	33.44	6539	66.56			617	43.09	788	56.91			851	32.66	1568	67.34			1096	31.76	2188	68.24			961	31.45	1995	68.55		
≥50	305	36.99	437	63.01			59	42.77	72	57.23			88	38.70	130	61.30			79	30.31	127	69.69			79	39.44	108	60.56		
**Husband’s education level**																														
No education	240	46.84	241	53.16	125.05	<0.001***	55	62.58	33	37.42	67.40	<0.001***	78	39.55	90	60.45	38.49	0.007***	67	45.79	76	54.21	29.75	0.017**	40	49.05	42	50.95	27.56	0.001**
Primary	3268	36.91	5082	63.09			835	48.25	779	51.75			891	37.89	1407	62.11			918	33.01	1669	66.99			624	31.12	1227	68.88		
Secondary	4148	30.67	8972	69.33			667	37.30	1182	62.70			937	30.36	2114	69.64			1338	28.89	3084	71.11			1206	30.09	2592	69.91		
Higher	851	34.47	1727	65.53			82	31.53	182	68.47			126	30.37	352	69.63			317	34.65	625	65.35			326	37.91	568	62.09		
**Husband’s occupation**																														
Not working	97	40.13	161	59.87	4.52	0.152	36	38.00	54	62.00	0.84	0.595	0	0.00	0	100.00			47	48.35	68	51.65	12.59	0.007***	14	27.72	39	72.28	0.29	0.646
Working	8409	33.49	15862	66.51			1603	42.66	2122	57.34			2031	20.32	3964	79.68			2593	30.90	5386	69.10			2182	31.52	4390	68.48		
**Diarrhea**																														
No	6459	35.41	11139	64.59	94.76	<0.001***	1372	45.91	1638	54.09	53.76	<0.001***	1714	36.26	2840	63.74	62.11	<0.001***	1907	32.08	3788	67.92	8.95	0.038*	1466	32.23	2873	67.77	3.11	0.165
Yes	2048	28.91	4883	71.09			267	32.30	538	67.70			318	24.85	1123	75.15			733	28.69	1666	71.31			730	30.12	1556	69.88		
**Fever**																														
No	3320	44.58	4156	55.42	605.08	<0.001***	557	53.18	511	46.82	66.80	<0.001***	894	45.81	1107	54.19	220.14	<0.001***	1239	41.80	1707	58.20	256.5	<0.001***	630	42.65	831	57.35	107.52	<0.001***
Yes	5187	28.57	11866	71.43			1082	38.52	1665	61.48			1138	26.90	2856	73.10			1401	24.75	3747	75.25			1566	28.36	3598	71.64		
**Acute respiratory infection**																														
No	6993	34.42	12525	65.58	37.63	<0.001***	1144	45.28	1370	54.72	27.98	0.003**	1412	34.78	2538	65.22	7.97	0.109	2421	31.95	4750	68.05	24.6	<0.001***	2016	32.45	3867	67.55	24.34	<0.001***
Yes	1514	29.62	3497	70.38			495	35.99	806	64.01			620	31.06	1425	68.94			219	23.51	704	76.49			180	23.25	562	76.75		
**Meeting expenses**																														
Big problem	2325	41.10	3126	58.90	158.61	<0.001***	604	52.82	503	47.18	55.37	<0.001***	842	42.68	1118	57.32	87.81	<0.001***	455	32.59	840	67.41	1.45	0.413	424	38.18	665	61.82	28.21	<0.001***
Not a big problem	6182	31.64	12896	68.36			1035	39.09	1673	60.91			1190	30.02	2845	69.98			2185	30.84	4614	69.16			1772	30.12	3764	69.88		
**Distance to nearest healthcare facility**																														
Big problem	1657	43.02	2098	56.98	147.55	<0.001***	381	56.64	276	43.36	46.52	<0.001***	559	41.97	700	58.03	39.53	<0.001***	393	38.77	640	61.23	27.23	<0.001***	324	40.78	482	59.22	35.3	<0.001***
Not big problem	6850	32.14	13924	67.86			1258	40.43	1900	59.57			1473	31.86	3263	68.14			2247	30.15	4814	69.85			1872	30.25	3947	69.75		
**Covered by health insurance**																														
No	2529	31.73	4811	68.27	1.65	0.376	N/A	N/A	N/A	N/A			N/A	N/A	N/A	N/A			1652	31.59	3176	68.41	1.57	0.431	877	31.99	1635	68.01	0.55	0.571
Yes	2307	30.75	5072	69.25			N/A	N/A	N/A	N/A			N/A	N/A	N/A	N/A			988	30.25	2278	69.75			1319	31.13	2794	68.87		
**Wealth index**																														
Poorest	3199	41.44	4211	58.56	218.65	<0.001***	730	58.74	487	41.26	158.18	<0.001***	853	43.88	1063	56.12	109.49	<0.001***	931	37.02	1457	62.98	38.11	0.003***	685	34.74	1204	65.26	24.69	0.009**
Poorer	1721	34.12	3409	65.88			338	47.20	450	52.80			418	35.73	824	64.27			528	30.90	1189	69.10			437	29.37	946	70.63		
Middle	1410	31.09	3050	68.91			227	38.46	405	61.54			302	29.42	752	70.58			452	28.93	1034	71.07			429	31.04	859	68.96		
Richer	1210	29.62	2932	70.38			196	33.21	434	66.79			260	29.46	706	70.54			409	29.44	983	70.56			345	27.91	809	72.09		
Richest	967	30.28	1420	69.72			148	31.70	400	68.30			199	26.74	618	73.26			320	28.61	-209	71.39			300	35.23	611	64.77		
**Place of residence**																														
Urban	3457	33.29	7069	66.71	0.68	0.63	530	37.02	1070	62.98	44.51	<0.001***	634	32.19	1534	67.81	3.87	0.279	1196	31.88	2414	68.12	2.19	0.391	1097	33.76	2051	66.24	13.85**	0.006**
Rural	5050	33.78	8953	66.22			1109	47.71	1106	52.29			1398	34.63	2429	65.37			1444	30.35	3040	69.65			1099	29.50	2378	70.50		
**Region of residence**																														
Eastern Indonesia	683	35.76	1164	64.24	56.22	<0.001***	N/A	N/A	N/A	N/A			209	35.33	338	64.67	5.23	0.151	268	36.76	431	63.24	26.22	<0.001***	206	35.11	395	64.89	27.77	<0.001***
Central Indonesia	3203	38.22	5100	61.78			655	46.91	710	53.09	6.35	0.027*	752	36.37	1328	63.63			962	36.23	1705	63.77			834	37.67	1357	62.33		
Western Indonesia	4621	32.42	9758	67.58			984	41.61	1466	58.39			1071	32.90	2297	67.10			1410	29.76	3318	70.24			1156	29.97	2677	70.03		

*p < 0.05; **p < 0.01; ***p < 0.00

### Multivariate analysis

Table 3 depicts the results of the multivariate analysis (logistic regression) of factors associated with healthcare-seeking behavior. Diseases among children under five, such as diarrhea, fever, and ARI symptoms, showed a statistically significant relationship with mothers’ engagement in healthcare-seeking behaviors. For example, mothers of children with fever had 2.25-times higher odds of seeking healthcare than those of children who had no signs of fever (OR = 2.25; 95% CI = 2.05–2.46). Meanwhile, mothers of children who experienced ARI symptoms had 1.43-times greater odds of seeking healthcare when compared to those of children who had no ARI symptoms (OR = 1.43; 95% CI = 1.27–1.62).

**Table 3:** Multiple logistic regression analysis of mothers’ healthcare-seeking behaviors for their children aged 0–59 months in Indonesia (prepared by the authors from analysis of the data).

Variables	2002			2007			2012			2017			2002–2017 (TOTAL)		
	AOR	95%CI		AOR	95%CI		AOR	95%CI		AOR	95%CI		AOR	95%CI	
		Lower	Upper		Lower	Upper		Lower	Upper		Lower	Upper		Lower	Upper
**Year**															
2017													1.57***	1.35	1.81
2012													1.80***	1.56	2.09
2007													1.62***	1.39	1.90
2002–3													1.00		
**Child’s age (months)**															
<12	1.08	0.70	1.67	1.56**	1.18	2.05	1.28*	1.03	1.58	1.23*	1.03	1.48	1.30***	1.14	1.47
12–36	1.00	0.70	1.43	1.18	0.94	1.49	1.20*	1.00	1.42	1.32***	1.14	1.53	1.20***	1.09	1.32
>36	1.00			1.00			1.00			1.00					
**Birth order**															
1^st^	1.29	0.89	1.87	1.19	0.88	1.60	1.24*	1.01	1.52	0.96	0.97	1.20	1.17*	1.03	1.33
2^nd^–3^rd^	1.16	0.83	1.61	1.16	0.90	1.51	1.20	0.98	1.45	0.99	0.95	1.21	1.15*	1.02	1.29
≥4^th^	1.00			1.00			1.00			1.00			1.00		
**Mother’s education level**															
No education	1.00			1.00			1.00			1.00			1.00		
Primary	0.96	0.48	1.91	1.48	0.95	2.30	0.94	0.59	1.52	1.75	0.97	3.14	1.23	0.93	1.64
Secondary	1.20	0.59	2.45	1.60	1.00	2.58	1.02	0.63	1.65	1.72	0.95	3.09	1.35*	1.01	1.81
Higher	1.91	0.76	4.77	1.75	0.91	3.36	0.84	0.48	1.46	1.42	0.76	2.67	1.17	0.84	1.63
**Mother’s occupation**															
Not working	1.11	0.84	1.45	1.06	0.87	1.30	1.00	0.87	1.15	1.07	0.92	1.24	1.09	0.99	1.18
Working	1.00			1.00			1.00			1.00			1.00		
**Husband’s education level**															
No education	1.00			1.00			1.00			1.00			1.00		
Primary	1.25	0.46	3.36	0.92	0.55	1.55	1.37	0.85	2.20	1.78	0.94	3.34	1.21	0.88	1.67
Secondary	1.46	0.52	4.08	1.09	0.64	1.84	1.61	0.99	2.63	1.83	0.96	3.48	1.38	1.00	1.92
Higher	1.42	0.43	4.65	0.88	0.45	1.74	1.34	0.75	2.41	1.53	0.77	3.04	1.16	0.80	1.68
**Husband’s occupation**															
Not working	1.19	0.53	2.67	N/A	N/A	N/A	0.50	0.29	0.85	1.29	0.62	2.68	0.87	0.57	1.31
Working	1.00			N/A	N/A	N/A	1.00			1.00			1.00		
**Diarrhea**															
No	1.00			1.00			1.00			1.00			1.00		
Yes	1.84***	1.39	2.44	1.65***	1.31	2.08	1.38***	1.18	1.61	1.57***	1.34	1.86	1.49***	1.36	1.64
**Fever**															
No	1.00			1.00			1.00			1.00			1.00		
Yes	1.89***	1.48	2.43	2.41***	1.97	2.95	2.34***	2.04	2.69	2.45***	2.05	2.93	2.25***	2.05	2.46
**Acute respiratory infection**															
No	1.00			1.00			1.00			1.00			1.00		
Yes	1.55**	1.17	2.05	1.18	0.95	1.46	1.53***	1.21	1.93	1.95***	1.53	2.48	1.43***	1.27	1.62
**Meeting expenses**															
Big problem	1.00			1.00			1.00			1.00			1.00		
Not a big problem	1.37	0.98	1.90	1.59***	1.28	1.97	0.90	0.72	1.12	1.28*	1.04	1.56	1.28***	1.14	1.44
**Distance to nearest healthcare facility**															
Big problem	1.00			1.00			1.00			1.00			1.00		
Not a big problem	1.33	0.92	1.92	1.12	0.89	1.42	1.47**	1.16	1.86	1.45**	1.14	1.83	1.34***	1.18	1.53
**Wealth index**															
Poorest	1.00			1.00			1.00			1.00			1.00		
Poorer	1.42	0.95	2.12	1.36*	1.04	1.78	1.29*	1.04	1.59	1.18	0.96	1.45	1.27***	1.11	1.43
Middle	2.13**	1.37	3.30	1.76***	1.32	2.34	1.46**	1.16	1.84	1.15	0.92	1.43	1.48***	1.29	1.70
Richer	2.22**	1.41	1.41	1.80***	1.25	1.58	1.51**	1.18	1.95	1.40**	1.11	1.75	1.61***	1.38	1.87
Richest	2.40**	1.37	1.37	2.35***	1.60	3.46	1.79***	1.32	2.44	1.15	0.87	1.53	1.78***	1.48	2.13
**Place of residence**															
Urban	1.00			1.00			1.00			1.00			1.00		
Rural	1.06	0.78	1.43	1.24	0.96	1.59	1.31**	1.10	1.57	1.33***	1.15	1.55	1.24***	1.12	1.37
**Region of residence**															
Eastern Indonesia	N/A	N/A	N/A	1.13	0.81	1.56	1.23	0.88	1.74	1.27	0.94	1.72	1.27*	1.03	1.55
Central Indonesia	1.00			1.00			1.00			1.00			1.00		
Western Indonesia	0.96	0.76	1.21	1.02	0.84	1.24	1.24**	1.07	1.43	1.40***	1.20	1.62	1.18*	1.08	1.28

*p < 0.05; **p < 0.01; ***p < 0.00

AOR, Adjusted odds ratio; 95% CI, 95% confidence interval.

Based on the data, mothers who have children aged <12 months are more likely to seek healthcare services (OR = 1.30; 95% CI = 1.14–1.47) than mothers who have children aged 12–36 months (OR = 1.20; 95% CI = 1.09–1.32). In addition, odds of seeking healthcare services are higher when the child is a first child (OR = 1.17; 95% CI = 1.03–1.33) than a second or third child (OR = 1.15; 95% CI = 1.02–1.29).

In addition, household socioeconomic factors were found to be significantly associated with healthcare-seeking behaviors. For example, mothers with secondary-level education had 1.35-times higher odds of seeking healthcare (OR = 1.35, 95% CI = 1.01–1.81), households in the richest quintile had 1.78-times higher odds (OR = 1.78; 95% CI = 1.48–2.13), and households that had no difficulties meeting expenses had 1.28-times higher odds (OR = 1.28; 95% CI = 1.14–1.44). Meanwhile, the findings revealed that families with no problems regarding the distance to the nearest healthcare facility had 1.34-times higher odds (OR = 1.34; 95% CI = 1.18–1.53) of seeking healthcare for children under five.

Moreover, households’ demographic factors, including place and region of residence, were determined to be associated with engagement in healthcare-seeking behaviors for children aged 0–59 months in Indonesia. For example, respondents who lived in rural areas had 1.24-times higher odds of engaging in healthcare-seeking behaviors than were respondents who lived in urban areas. Additionally, respondents who lived in Eastern Indonesia had 1.27-times higher odds than those who lived in Western and Central Indonesia.

## Discussion

Considering the relatively high under-five mortality rate in Indonesia, the present study aimed to analyze the determinants of mothers’ engagement in healthcare-seeking behaviors for children under five. Using nationally representative data for 2002–2017, we explored various factors, including socioeconomic and demographic factors, related to mothers’ engagement in healthcare-seeking behaviors. Our findings showed that the presence of symptoms of childhood diseases, such as diarrhea, fever, and ARI, significantly affect the likelihood of mothers in Indonesia engaging in healthcare-seeking behaviors for their children. This study also revealed that child, socioeconomic, and demographic factors, such as child’s age, ability to meet expenses, accessibility of healthcare facilities, wealth index, and place and region of residence, were significantly associated with engagement in healthcare-seeking behavior.

Our study found that mothers of children with fever and/or ARI symptoms are more likely to seek healthcare services than mothers of children who do not experience these common childhood diseases. Previous studies have revealed that caregivers of children who experience ARI symptoms are more likely to seek healthcare for their child (70.4%) when compared to those of children who experience fever (68.5%) and children who experience diarrhea (63.3%), respectively [11]. Consistent with these findings, other studies conducted in developing countries have reported that children under five who have fever and ARI symptoms are more likely to be treated at healthcare facilities, whereas those with diarrhea are usually initially treated at home [21, 22]. Children with diarrhea may be treated effectively at home by caregivers through oral rehydration and zinc supplements; in contrast, ARI symptoms and fever indicate severe disease and vulnerability. Therefore, children who experience these latter symptoms require healthcare from professional providers and immediate antibiotic treatment. Enhance the awareness and skill of mothers in seeking health care for the sick children is needed.

We found that mothers who had children aged <12 months were more likely to seek healthcare than mothers who had older children (>36 months). Similar findings have been obtained in previous studies, with some studies reporting that mothers of older children are less likely to seek healthcare than those of younger children [15, 22, 23], and other studies reporting that mothers of children under 12 months of age are more likely to bring their children to healthcare facilities and obtain medical treatment than those with children aged over 12 months [21, 24–26]. A possible reason for this is the vulnerability of younger children aged <12 months due to their immature immune systems and relatively high risk of infection from peers.

Our study found that mothers are more likely to seek healthcare for their first child than their second or third children. Possible reasons for this include the fact that one’s first child may be more prone to illness, and novice mothers may have incorrect conceptions regarding child health. Notably, an earlier study reported that older children receive less attention in regard to healthcare-seeking because of their maturity [27]. Commonly, mothers pay more attention to and have greater awareness of their first child’s health. Once the first child is diagnosed with an illness, mothers tend to take immediate action to seek healthcare. Mothers with two and three children are likely to have more experience; thus, they are less likely to seek medical attention for problems regarding their children’s health. Routine visits to health facilities need to be scheduled to prevent illness in children.

We found that women with secondary education are more likely to seek healthcare for their children than are those with no educational background. Thus, mothers’ knowledge levels may play a role in their decisions whether to use the healthcare system and whether to delay receiving professional care. This is consistent with other findings for low- and middle-income countries, which have indicated the role of women’s education in regard to their engagement in healthcare-seeking for their children [24, 28, 29]. Some study also found that women with higher levels of education are more likely to seek health care services for their children [30, 31]. Continuous effort to increase the access into education especially for Indonesian women is crucial in the long run.

The present study showed that mothers who reported that meeting expenses was not a big problem were more likely to seek healthcare for their children. The role of difficulty meeting expenses in this regard has been reported in a previous study, which stated that, if money is a notable issue for families, children who have fever and coughs are less likely to receive healthcare [32]. Notably, in Ethiopia, where healthcare is offered at minimum cost in rural areas, the additional costs associated with transportation and treatment can create difficulties for households [32]. In Indonesia, a national health insurance system (NHI) has been implemented across the country; however, inequalities remain, particularly in regard to low-income families, informal workers, and families with children under four years of age [33]. NHI membership coverage data for 2016 shows that only about 63% of Indonesian people have NHI [34]. Our study results also show that more than half do not have health insurance. Thus, families’ financial status could affect their decisions to seek healthcare and travel to healthcare facilities.

Furthermore, this study revealed that mothers who reported that the distance to the nearest healthcare facility was not a big issue were more likely to seek healthcare for their children. This finding is supported by those of previous studies conducted in other countries [13, 15, 35]. Distance to a healthcare facility might be a common influencing factor underlying mothers’ engagement in healthcare-seeking behaviors as a result of the efforts and transportation costs required to reach healthcare facilities. Although public-health facilities are available in each local community across Indonesia, empirical evidence has revealed that distance to healthcare facilities remains a significant challenge for obtaining healthcare [36]. Especially in the context of Indonesia, which is the largest archipelagic country in the world, this has resulted in uneven access to roads and transportation to health facilities in all regions.

This study found that richer households are more likely than poorer households to seek healthcare at healthcare facilities. This finding is consistent with those of other studies that have shown that more affluent families, when compared to low-income families, are more likely to obtain medical treatments for their children [24, 36, 37]. Thus, it is apparent that wealth index plays an essential role in mothers’ engagement in healthcare-seeking behavior [24]. A possible reason for this is that households with greater incomes can afford to pay for health care and medical insurance [38], making them more likely to bring their children to medical facilities. Meanwhile, parents from the poorest quintiles are likely to experience more daily life burdens, making it more difficult to find the money to seek healthcare for their children. In addition, traditional medicine and self-medication are still prominent in Indonesian culture, such as making traditional herbal medicine and performing massage to cure illness [39]. Some of these traditional treatments were chosen because they are low cost and almost never require payment, making them more accessible to poorer households.

Our study found that place and region of residence are associated with mothers’ engagement in healthcare-seeking behaviors for children aged 0–59 months in Indonesia. Mothers who live in rural areas are more likely to seek medical care for children than are mothers who live in urban areas. This finding is also consistent with those of other studies, which have shown that rates of care-seeking are higher in rural areas than in urban areas [13, 40]. A possible reason for this is that mothers in urban areas have better health conditions and nutrition, which influences the nutritional status of their children and reduces the risk of childhood diseases. Another possibility is that mothers who live in urban areas assume that they have adequate knowledge and skills for managing common childhood diseases, which could, therefore, reduce their likelihood of seeking healthcare. A qualitative study conducted in urban areas in Indonesia also stated that the majority of families prefer traditional ways of caring for their sick children, such as scraping with shallots and other traditional medicines. In Indonesia there is also a spiritual belief against the prohibition of the use of medical treatment [41, 42].

Additionally, mothers living in Eastern and Western Indonesia were found to be more likely to seek medical care for their children than mothers living in Central Indonesia. The western region of Indonesia has more developed regions than the eastern and central regions, with the latter two regions being generally known as underdeveloped areas with significant poverty rates and lower living standards when compared to the western region [43]. Empirical evidence shows that socio-economic development, including transportation, roads, and healthcare facilities, is not evenly distributed throughout Indonesia, especially in the eastern region [44–46]. Further, the majority of doctors in Indonesia (57.4%) are located on Java, which is part of the western region [47]. Studies have indicated that easy access to healthcare facilities and adequate numbers of healthcare workers can increase mothers’ likelihood of seeking healthcare for their children [48, 49]. This may be one of the reasons mothers who live in the western region of Indonesia show greater healthcare-seeking behaviors for their children than those in the central region. Surprisingly, however, our findings show that mothers who live in the eastern region have the highest tendency to seek healthcare services for their children. People living in eastern Indonesia experience a much higher rate of disruptive morbidity than those living in western Indonesia [50]. In 2004, almost one-fifth of people living in eastern Indonesia experienced disruptive sickness, causing them to use healthcare facilities more often [51]. Although, in Indonesia, as an archipelagic country, geographical constraints can impact the availability and affordability of health services for families, especially those in the Eastern region, based on our findings mothers in this region still prioritize seeking health for their children.

To our knowledge, this study is the first to use data from the 2002–2017 IDHS to explore the determinants of engagement in healthcare-seeking behaviors among mothers in Indonesia for children aged under five. Our study examined various factors affecting mothers’ engagement in healthcare-seeking behavior for children of this age group. However, this study, nevertheless, contains several limitations. This study used a cross-sectional method and, therefore, causality could not be determined. Moreover, although the analysis for this study fully utilized for the available data from the IDHS, the dataset featured limited coverage of predictor variables; thus, the predictor factors were not analyzed because of unavailability in the dataset. In addition, the survey was based solely on mothers’ responses (self-reported) two weeks preceding the study; therefore, recall bias may be present. Despite these limitations, however, our study has a strength in that it used a dataset featuring the most recent nationally and provincially representative data obtained through a survey that had a high response rate (>95%). Moreover, these data were nationally and internationally standardized. The uniqueness of this study in terms of healthcare seeking practices of mothers for their sick children was mothers remains at the forefront of care. Empowering mother with supportive resources especially at the community level contribute to the positive impact on children’s health outcomes in Indonesia.

## Conclusions

Several factors are related to mothers’ engagement in healthcare-seeking behavior for children under the age of five in Indonesia. Symptoms of common childhood diseases, such as diarrhea, fever, and ARI, are significantly associated with such engagement in healthcare-seeking behaviors. Additionally, child’s age, birth order, mother’s education level, ability to meet expenses, distance to the nearest healthcare facility, wealth index, and place and region of residence were determined to significantly influence such engagement. Our findings reveal that it remains necessary to promote public awareness of childhood diseases and the importance of seeking healthcare services when children are ill. Moreover, our study has policy implications: we suggest that, for families who are socioeconomically and geographically disadvantaged, healthcare facilities be made readily available, approachable, and affordable. Moreover, regarding future research, several factors influence the barriers and facilitators healthcare professionals experience when seeking to deliver healthcare services; therefore, qualitative research should be undertaken to determine the health-service preferences of mothers and families, as this information could help address such barriers.

## References

[1] Paulson KR, Kamath AM, Alam T, Bienhoff K, Abady GG, Abbas J, et al. Global, regional, and national progress towards Sustainable Development Goal 3.2 for neonatal and child health: all-cause and cause-specific mortality findings from the Global Burden of Disease Study 2019. Lancet. 2021;398(10303):870–905. doi: 10.1016/S0140-6736(21)01207-1 34416195 PMC8429803

[2] WHO. Child Mortality/Causes of Death [Internet]. Geneva, Swiss: WHO; 2020. Available from: https://www.who.int/data/maternal-newborn-child-adolescent-ageing/child-data/child—mortality-causes-of-death.

[3] WHO. Children: Improving Survival and Well-Being [Internet]. WHO. 2020 [cited 2021 Feb 21]. Available from: https://www.who.int/news-room/fact-sheets/detail/children-reducing-mortality.

[4] United Nations. SDGs Indicators [Internet]. 2021 [cited 2021 Feb 20]. Available from: https://unstats.un.org/sdgs/metadata/?Text=&Goal=3&Target=3.2.

[5] Warrohmah ANINI, Berliana SMMSM Nursalam N, Efendi F, Haryanto J, Has EMMMM, et al. Analysis of the Survival of Children under Five in Indonesia and Associated Factors. In: M.A. R, editor. 3rd International Conference on Tropical and Coastal Region Eco Development 2017. Statistics Indonesia (BPS), Lampung, Kota Metro, Indonesia: Institute of Physics Publishing; 2018.

[6] Ministry of Health Republic Indonesia. Indonesia Health Profile. Jakarta, Indonesia: Ministry of Health Republic Indonesia; 2018.

[7] UNICEF. UNICEF Data: Moitoring The Situation of Children and Women [Internet]. UNICEF. 2020 [cited 2021 Feb 20]. Available from: https://data.unicef.org/country/mys/.

[8] Windi R, Efendi F, Qona’ah A, Adnani QES, Ramadhan K, Almutairi WM. Determinants of Acute Respiratory Infection Among Children Under-Five Years in Indonesia. J Pediatr Nurs. 2021;60:e54–9. doi: 10.1016/j.pedn.2021.03.010 33744057

[9] BKKBN BPS, Kemenkes ICF. Indonesia Demographic and Health Survey 2017. Jakarta, Indonesia: BKKBN, BPS, Kemenkes, ICF; 2018.

[10] Kemenkes RI. Profil Kesehatan Indonesia Tahun 2019. Jakarta, Indonesia: Kemenkes Republik Indonesia; 2020.

[11] Bradley SEK, Rosapep L, Shiras T. Where Do Caregivers Take Their Sick Children for Care? An Analysis of Care Seeking and Equity in 24 USAID Priority Countries. Glob Heal Sci Pract. 2020 Sep;8(3):518–33. doi: 10.9745/GHSP-D-20-00115 33008861 PMC7541105

[12] Geldsetzer P, Williams TC, Kirolos A, Mitchell S, Ratcliffe LA, Kohli-Lynch MK, et al. The Recognition of and Care Seeking Behaviour for Childhood Illness in Developing Countries: A Systematic Review. PLoS One [Internet]. 2014 Apr 9;9(4):e93427. Available from: doi: 10.1371/journal.pone.0093427 24718483 PMC3981715

[13] Arthur E. The Effect of Household Socioeconomic Status on the Demand for Child Health Care Services. African Dev Rev [Internet]. 2019 Mar 1;31(1):87–98. Available from: 10.1111/1467-8268.12365

[14] Colvin CJ, Smith HJ, Swartz A, Ahs JW, de Heer J, Opiyo N, et al. Understanding careseeking for child illness in sub-Saharan Africa: a systematic review and conceptual framework based on qualitative research of household recognition and response to child diarrhoea, pneumonia and malaria. Soc Sci Med. 2013 Jun;86:66–78. doi: 10.1016/j.socscimed.2013.02.031 23608095

[15] Bennett A, Eisele T, Keating J, Yukich J. Global trends in care seeking and access to diagnosis and treatment of childhood illnesses. DHS Work Pap [Internet]. 2015;(116):iv-pp. Available from: http://dhsprogram.com/pubs/pdf/WP116/WP116.pdf

[16] Mboi N, Syailendrawati R, Ostroff SM, Elyazar IR, Glenn SD, Rachmawati T, et al. The state of health in Indonesia’s provinces, 1990–2019: a systematic analysis for the Global Burden of Disease Study 2019. Lancet Glob Heal. 2022;10(11):e1632–45. doi: 10.1016/S2214-109X(22)00371-0 36240829 PMC9579357

[17] DHSProgram. Wealth Index Rockville [Internet]. 2016 [cited 2021 Feb 20]. Available from: https://dhsprogram.com/topics/wealth-index/.

[18] Hartojo N, Ikhsan M, Dartanto T, Sumarto S. A Growing Light in the Lagging Region in Indonesia: The Impact of Village Fund on Rural Economic Growth. Economies. 2022;10(9).

[19] STATA. Svyset-Declare survey design for dataset. Texas: Stata; 2022.

[20] Croft TN, Marshall AMJ, Allen CK. Guide to DHS Statistics. Rockville, Maryland, USA: ICF. Rockville, Maryland, USA: ICF International; 2018. 22–51 p.

[21] Kanté AM, Gutierrez HR, Larsen AM, Jackson EF, Helleringer S, Exavery A, et al. Childhood Illness Prevalence and Health Seeking Behavior Patterns in Rural Tanzania. BMC Public Health [Internet]. 2015;15(1):951. Available from: doi: 10.1186/s12889-015-2264-6 26399915 PMC4581421

[22] Lungu EA, Darker C, Biesma R. Determinants of healthcare seeking for childhood illnesses among caregivers of under-five children in urban slums in Malawi: a population-based cross-sectional study. BMC Pediatr [Internet]. 2020;20(1):20. Available from: doi: 10.1186/s12887-020-1913-9 31952484 PMC6966883

[23] Ng’ambi W, Mangal T, Phillips A, Colbourn T, Mfutso-Bengo J, Revill P, et al. Factors associated with healthcare seeking behaviour for children in Malawi: 2016. Trop Med Int Health. 2020 Dec;25(12):1486–95. doi: 10.1111/tmi.13499 32981174

[24] Sabbir Ahmed M, Yunus FM. Healthcare seeking behavior for common illness among Bangladeshi under-five children: a nationwide cross-sectional survey. Child Youth Serv Rev [Internet]. 2020;119:105644. Available from: https://www.sciencedirect.com/science/article/pii/S0190740920320673

[25] Abegaz NT, Berhe H, Gebretekle GB. Mothers/caregivers healthcare seeking behavior towards childhood illness in selected health centers in Addis Ababa, Ethiopia: a facility-based cross-sectional study. BMC Pediatr [Internet]. 2019;19(1):220. Available from: doi: 10.1186/s12887-019-1588-2 31269920 PMC6607537

[26] Abdulkadir MB, Abdulkadir ZA. A cross-sectional survey of parental care-seeking behavior for febrile illness among under-five children in Nigeria. Alexandria J Med [Internet]. 2017 Mar 1;53(1):85–91. Available from: 10.1016/j.ajme.2016.02.005

[27] Adedokun ST, Yaya S. Factors influencing mothers’ health care seeking behaviour for their children: evidence from 31 countries in sub-Saharan Africa. BMC Health Serv Res. 2020 Sep;20(1):842. doi: 10.1186/s12913-020-05683-8 32894107 PMC7487813

[28] Sultana M, Sarker AR, Sheikh N, Akram R, Ali N, Mahumud RA, et al. Prevalence, determinants and health care-seeking behavior of childhood acute respiratory tract infections in Bangladesh. PLoS One [Internet]. 2019 Jan 10;14(1):e0210433. Available from: 10.1371/journal.pone.0210433 PMC6328134 30629689

[29] Pinzón-Rondón ÁM, Aguilera-Otalvaro P, Zárate-Ardila C, Hoyos-Martínez A. Acute respiratory infection in children from developing nations: a multi-level study. Paediatr Int Child Health. 2016 May;36(2):84–90. doi: 10.1179/2046905515Y.0000000021 25936959

[30] Yaya S, Odusina EK, Adjei NK. Health care seeking behaviour for children with acute childhood illnesses and its relating factors in sub-Saharan Africa: evidence from 24 countries. Trop Med Health. 2021;49(1):1–8. 34906263 10.1186/s41182-021-00385-1 PMC8670049

[31] Kagabo DM, Kirk CM, Bakundukize B, Hedt-Gauthier BL, Gupta N, Hirschhorn LR, et al. Care-seeking patterns among families that experienced under-five child mortality in rural Rwanda. PLoS One. 2018;13(1):e0190739. doi: 10.1371/journal.pone.0190739 29320556 PMC5761861

[32] Pierce H, Gibby AL, Forste R. Caregiver Decision-Making: Household Response to Child Illness in sub-Saharan Africa. Popul Res Policy Rev. 2016 Oct;35(5):581–97. doi: 10.1007/s11113-016-9396-y 28794575 PMC5546145

[33] Agustina R, Dartanto T, Sitompul R, Susiloretni KA, Suparmi, Achadi EL, et al. Universal health coverage in Indonesia: concept, progress, and challenges. Lancet (London, England). 2019 Jan;393(10166):75–102. doi: 10.1016/S0140-6736(18)31647-7 30579611

[34] Dewan Jaminan Sosial Nasional BPJS Kesehatan. Statistik JKN 2014–2018: mengungkap fakta dengan data [Internet]. 2020. Available from: https://djsn.go.id/files/dokumen/DokumenKajian/202104151516StatistikJKN2014–2018_PDFE-Book_CetakanPertama(DJSN—BPJSKesehatan).pdf

[35] Rutherford ME, Mulholland K, Hill PC. How access to health care relates to under-five mortality in sub-Saharan Africa: systematic review. Trop Med Int Health. 2010 May;15(5):508–19. doi: 10.1111/j.1365-3156.2010.02497.x 20345556

[36] Titaley CR, Que BJ, de Lima FVI, Angkejaya OW, de Lima FVI, Maelissa MM, et al. Health Care-Seeking Behavior of Children With Acute Respiratory Infections Symptoms: Analysis of the 2012 and 2017 Indonesia Demographic and Health Surveys. Asia-Pacific J public Heal. 2020;32(6–7):310–9. doi: 10.1177/1010539520944716 32729324

[37] Ayalneh AA, Fetene DM, Lee TJ. Inequalities in health care utilization for common childhood illnesses in Ethiopia: evidence from the 2011 Ethiopian Demographic and Health Survey. Int J Equity Health [Internet]. 2017;16(1):67. Available from: doi: 10.1186/s12939-017-0561-7 28431502 PMC5399816

[38] Woolf SH, Aron L, Dubay L, Simon SM, Zimmerman E, Luk KX. How are Income and Wealth Linked to Health and Longevity? The Gradient between Economic Wellbeing and Health. Urban Inst. 2015;1(April):1–21.

[39] Pengpid S, Peltzer K. Use of traditional medicines and traditional practitioners by children in Indonesia: Findings from a national population survey in 2014–2015. J Multidiscip Healthc. 2019;12:291–8. doi: 10.2147/JMDH.S203343 31114218 PMC6497110

[40] Hodgins S, Pullum T, Dougherty L. Understanding where parents take their sick children and why it matters: a multi-country analysis. Glob Heal Sci Pract. 2013 Nov;1(3):328–56. doi: 10.9745/GHSP-D-13-00023 25276548 PMC4168586

[41] Kurniawan A. Health-Seeking Behavior (a Phenomenological Study on Health-Seeking Behavior Among the Parents With Under-Five Age Children in Sumpiuh Sub District). J Anal Sosiol. 2022;11(3):556–73.

[42] Purwati NH, Rustina Y, Supriyatno B. Knowledge and healthcare-seeking behavior of family caregivers of children with pneumonia: A qualitative study in an urban community in Indonesia. Belitung Nurs J. 2021;7(1):107–12. 10.33546/bnj.1268 PMC10353579 37469949

[43] Sihombing P. Does the Gap Between East and West Still Exist? a Study of Indonesia’s Disparities. Udayana J Soc Sci Humanit. 2019 Mar 1;3:1.

[44] Mubasyiroh R, Nurchotimah E, Laksono A. Indeks Aksesibilitas Pelayanan Kesehatan di Indonesia. In 2016. p. 21–57.

[45] Bappenas. Prakarsa pemerintah daerah dalam upaya pengurangan kesenjangan wilayah dan pembangunan daerah. Jakarta, Indonesia: Bappenas; 2018.

[46] Cameron L, Contreras Suarez D, Cornwell K. Understanding the determinants of maternal mortality: An observational study using the Indonesian Population Census. PLoS One. 2019;14(6):e0217386. doi: 10.1371/journal.pone.0217386 31158243 PMC6546237

[47] Mahendradhata Y, Trisnantoro L, Listyadewi S, Soewondo P, Marthias T, Harimurti P, et al. The Republic of Indonesia health system review [Internet]. Vol. 7, Health Systems in Transition. New Delhi PP—New Delhi: WHO Regional Office for South-East Asia; 2017. Available from: https://apps.who.int/iris/handle/10665/254716

[48] Badolo H, Bado AR, Hien H, Méda N, Susuman AS. Factors associated with mothers’ health care-seeking behaviours for childhood fever in Burkina Faso: findings from repeated cross-sectional household surveys. Glob Heal Res Policy [Internet]. 2022;7(1):37. Available from: doi: 10.1186/s41256-022-00270-2 36266714 PMC9585735

[49] Merkeb Alamneh Y, Getachew M, Atnaf A, Abebaw A. Mothers’ health care-seeking behavior and associated factors for common childhood illnesses in Ethiopia: A systematic review and meta-analysis. SAGE Open Med [Internet]. 2022 Jan 1;10:20503121221099020. Available from: doi: 10.1177/20503121221099019 35615524 PMC9125608

[50] Laksono AD, Wulandari RD, Efendi F. Determinants of hospital utilisation among urban poor societies in Indonesia. Int J Innov Creat Chang. 2020;12(9):375–87.

[51] Suryadarma D, Widyanti W, Suryahadi A, Sumarto S. From Access to Income: Regional and Ethnic Inequality in Indonesia. SMERU Work Pap. 2006;(May 2006):ii–23.

